# Annotations

**Published:** 1894-06-09

**Authors:** 


### ANNOTATIONS.

### Sir Edward Sieveking on Out-patients.

Sir Edward Sieveking, consulting physician to St. Mary's Hospital, has contributed a brief but practical paper to the *British Medical Journal* on the burning question of out-patient departments at general hospitals. Sir Edward's contentions are, first, that the out-patient departments, as now conducted, are mere dispensaries; that as the result of such a condition of things patients are hurriedly treated, physicians are overworked, students make little real use of the departments, and that the whole thing is a scramble, an offence, and a degradation. Secondly, that the true and proper function of the out-patient department is one of consultation and teaching in specially selected cases. If these principles are adopted out-patient physicians will have time to do their work thoroughly, and the out-patient clinic will become quite as valuable as the in-patient; medical students will treat out-patients with scientific seriousness and thoroughness, and will thus devote much time to practical study, which they now devote to supercilious dawdling. Moreover, they will also learn the art of behaving towards their patients with that considerateness, which is due to every human being, especially to every sick human being; and they will find that they have learned a most valuable art when they begin to practice medicine on their own account. A third, and most desirable result will be that probably three-fourths of those who now seek the out-patient department without justification either in the nature of their illness or the necessitousness of their circumstances, will be cut off, and compelled to pay the ordinary practitioner for his services. There is no doubt that Sir Edward Sieveking's proposals would effect exactly the reform which is needed in the out-patient department, and in so doing would conserve the resources of hospitals, add to the dignity of their physicians and surgeons, and immensely soothe the mind and comfort the pocket of the hard-pressed general practitioner.

### Dermatology and General Medicine.

A clear thinker and a bold speaker is Dr. Philip Henry Pye-Smith. Clear thought and bold speech are things to be thankful for in medicine: to be thankful for, and to be imitated. "Never again," says he, "never again, we hope, will dermatology fall into the hands of empirics or specialists in the offensive sense of the word. It will be studied as a branch of scientific medicine by those who are thoroughly trained in general pathology, diagnosis, and treatment. Obsolete synonyms, pedantic and unscholarly innovations, artificial and elaborate classifications, will be discarded, together with secret remedies and advertised nostrums of unknown composition .... There is no branch of medicine which is so instructive in the broad principles of pathology and therapeutics as that of diseases of the skin .... the affected parts are obvious and easily investigated. We can see the various stages of disease, which are invisible in the case of mucous membranes, the solid viscera, or the bones and joints." There spoke the man who has recognised that the eyes are to see with, and the hands to handle with, and who has a heart-deep quarrel with most of the medicine of past ages, because it persistently refused to see with its eyes, and as resolutely insisted upon building logical forms upon purely imaginary data. To say that dermatology should be handed over to the specialist, and not studied by the practitioner of general medicine, would be ridiculous. Dermatology, of all the departments of medicine, is the very science to be studied by every practitioner, and by the student at the very beginning of his clinical career, and that for the reason given by Dr. Pye-Smith, that every anatomical fact can be seen, every pathological detail can be studied under the naked eye or with the lens in hand, and the results of every therapeutic method can be watched from day to day and from hour to hour. We congratulate the Dermatological Society of Great Britain and Ireland on the soundness of its constitutional principles, and on the bold breadth of its first public utterance by the mouth of its authorised representative and president.

### Small-pox and Vaccination at Leicester.

The recently-published report of Dr. Joseph Priestley, medical officer of health for the town of Leicester, is one evidence amongst many that a scientific training tends to produce a trustworthy reasonableness of mind. Medicine, whilst losing none of its faith in the positive value of vaccination, has learnt to understand better the limits of that value, and, moreover, has come to recognise that even a valuable method of prophylaxis is not to be thrust down the throat of a half-comprehending public at the point of the bayonet. Whilst, therefore, feeling that Leicester undertook an enormous responsibility in deciding to repudiate vaccination, a responsibility which facts have not justified her in assuming, we can yet feel a certain amount of satisfaction, from a scientific point of view, in the object lessons, on a considerable scale, with which she has furnished us on the question of vaccination as against non-vaccination. The value of those object lessons is greatly enhanced by the scientific exactness and fairmindedness with which they are described by Dr. Priestley. In brief, there has been no evidence of a visitation of poetical retributive justice against Leicester for refusing the benefits of vaccination, such as many enthusiastic believers in Jennerism looked for and perhaps hoped for. But for all that there has been enacted a series of experiences which are even more convincing to the sober and rational mind. A mild epidemic of small-pox prevailed in Leicester from August, 1892, until the close of 1893. In other towns almost all young children, by virtue of their recent vaccination, have escaped infection. But in Leicester, because of their non-vaccination, it is mostly young children which have been attacked. Moreover, of 107 children attacked, only two had been vaccinated; the remaining 105 were unvaccinated. Further, of those vaccinated children attacked, not only did none die, but none had the disease severely. Of the unvaccinated, 45 were severely attacked, 43 very severely, and 15 died. Some of the cases were accompanied by extravasions of blood in the skin, eyes, lungs, and elsewhere; their appearance was described as "revolting," and they were said to resemble the cases of "black" small-pox recorded by the writers of earlier days. These facts require no comment. What they do require is candid consideration by honest and open minds.

